# Editorial: Inflammation in ischemic heart disease: pathophysiology, biomarkers, and therapeutic implications

**DOI:** 10.3389/fcvm.2024.1469413

**Published:** 2024-08-15

**Authors:** Luigi Cappannoli, Mattia Galli, Josip Andelo Borovac, Emanuele Valeriani, Francesco Maria Animati, Francesco Fracassi, Francesco Burzotta

**Affiliations:** ^1^ Department of Cardiovascular Sciences, Università Cattolica del Sacro Cuore (UCSC), Rome, Italy; ^2^ Department of Cardiovascular Sciences, Fondazione Policlinico Universitario A. Gemelli IRCCS, Rome, Italy; ^3^ GVM Care & Research, Maria Cecilia Hospital, Cotignola, Italy; ^4^ Department of Medical-Surgical Sciences and Biotechnologies, Sapienza University of Rome, Latina, Italy; ^5^ Division of Interventional Cardiology, Cardiovascular Diseases Department, University Hospital of Split (KBC Split), Split, Croatia; ^6^ Department of General Surgery and Surgical Specialty, Sapienza University of Rome, Rome, Italy

**Keywords:** inflammation, ischemic heart disease, pathophysiology, target therapy, biomarkers

**Editorial on the Research Topic**
Inflammation in ischemic heart disease: pathophysiology, biomarkers, and therapeutic implications

In the Research Topic “*Inflammation in Ischemic Heart Disease: Pathophysiology, Biomarkers, and Therapeutic Implications*”, is provided a thorough and extensive overview about inflammation mechanisms leading to atherosclerotic plaque formation and consequent ischemic heart disease (IHD).

Inflammation plays a pivotal role in all stages of atherosclerotic disease. Atherosclerotic lesions are composed of immune system cells, connective tissue cells, lipids and debris. Among immune system cells, macrophages are involved in a transformation process which leads to the formation of foam cells, rich in low-density lipoproteins (LDL). After being phagocytosed by macrophages, LDL cholesterol undergoes an oxidative process which brings to oxidized LDLs (ox-LDLs), the main cause of atherosclerosis (1). The well-known Framingham Study was the first study to include cholesterol as a risk factor for ischemic heart disease, paving the way for subsequent landmark trials that demonstrated how the reduction of LDL cholesterol levels with statins significantly correlates with a lower risk of cardiovascular events (2). Other than lowering LDL levels, statins also play a pleiotropic effect that slows down the inflammatory processes underlying atherosclerotic disease (3). This was clearly assessed in the JUPITER study, that showed how statin treatment is effective not only in lowering LDLs levels, but also in C-reactive protein (CRP) reduction, reducing the risk of major cardiovascular events (MACEs) also in patients without hyperlipidemia (4).

Nevertheless, other factors interplay in atheroma formation. T-cells produce cytokines affecting both endothelial and smooth muscle cells functions, increasing the plaque volume and leading to its destabilization (5). In this perspective, atherosclerosis should be considered an inflammatory disease: immune responses interact with metabolic risk factors to start, spread, and activate lesions in the arteries. The role of immune system activation in IHD is corroborated by the elevation of some circulating biomarkers, such as myeloperoxidase, interleukins (IL), selectins (E-selectin, P-selectin), matrix metalloproteinases (MMPs), vascular adhesion molecule 1 (VCAM-1), tumor necrosis factor (TNF)-alpha, and CRP (6). In 1994, Liuzzo et al. were the first to show that elevated CRP and serum amyloid A protein levels in patients with unstable angina were associated with an increased occurrence of recurrent ischemia (7). These results are in line with the more recent PROVE-IT TIMI 22 trial and the already mentioned JUPITER trial. In the PROVE-IT TIMI 22 trial, patients with acute coronary syndrome (ACS) had better outcomes not only lowering LDL levels under 70 mg/dl, but also attaining low CRP concentration (below 2 mg/L) (8).

Although many biomarkers of atherosclerosis are already known, many others are still under investigation.

In the retrospective cohort study by Ma et al. included in this Research Topic, higher levels of systemic inflammation response index (SIRI) correlated with an increased risk of MACEs in 3,161 patients with heart failure who underwent percutaneous coronary intervention (PCI) after an ACS. The results of the study demonstrated that SIRI could represent an useful biomarker for predicting MACEs in patients with heart failure who undergo PCI. Another potential biomarker of IHD is IL-36. In the study by El-Awaisi et al., it was shown that IL-36 holds a fundamental role as a regulator of innate and adaptive immune responses in both acute and chronic coronary syndromes and that there is a significant elevation in IL-36 receptor and IL-36α/β subunits concentration in patients with ischemia-reperfusion injury, with greater levels in females. The same study demonstrated that treatment with IL-36 receptor antagonists significantly decreases neutrophil adhesion, increases functional capillary density and ventricular perfusion and reduces infarct size, oxidative damage and VCAM-1.

Inflammation pathways can be activated not only by endogenous molecules, but also by environmental factors. Recently, air pollution has emerged as an important risk factor for IHD, with PM2.5 and PM10 considered the most dangerous pollutants, since they can penetrate into distal airways, arriving in the bloodstream (9). PM2.5 is involved in activation of the nucleotide-binding oligomerization domain-like receptor protein 3 (NLRP3) inflammasome, thus activating to caspase-1 and enhancing the secretion of proinflammatory and proatherogenic cytokines. Moreover, it promotes the differentiation of macrophages toward a M1 phenotype, the oxidation of LDLs to ox-LDLs and it has been associated with increased levels of CRP, plaque instability and acute coronary syndromes (9).

Other studies correlated the concentration of inflammatory biomarkers with plaque characteristics assessed by intracoronary imaging techniques. A recent study showed that among patients suffering from an ACS, those with both high risk plaque features at optical coherence tomography (OCT) and CRP levels above 2 mg/L had a recurrent ACS during a 3-year follow-up (10).

The evidence of a fundamental role played by inflammation in IHD has brought to important therapeutic implications. In this regard, a remarkable result was given by the Canakinumab Anti-inflammatory Thrombosis Outcome Study (CANTOS) trial (11). Among 10,061 enrolled patients with high-sensitivity CRP (hs-CRP) values ≥2 mg/L, a significant benefit in terms of nonfatal myocardial infarction (MI), nonfatal stroke, cardiovascular death and hospitalization for unstable angina, was observed among those receiving Canakinumab (a monoclonal antibody against IL-1β), and with the maximal benefits in those who reported the most significant reduction of hs-CRP. Also the antinflammatory drug colchicine has been investigated as a potential therapeutic agent for IHD (12). Recently, the LoDoCo2 trial (Low-Dose Colchicine for secondary prevention of cardiovascular disease trial 2), which involved patients with stable IHD randomly assigned to receive either 0.5 mg of colchicine daily or conventional treatment, showed a significant reduction in the primary combined endpoint in the colchicine group (cardiovascular death, spontaneous MI, ischemic stroke, or ischemia-driven coronary revascularization), primarily due to a lower rate of ACS (13).

Lastly, little is still known about regenerative therapy for myocardium damaged by ischemic injury. In this context, the recent review by Xiao and Shi, enlightens the possible role of adipose-derived stem cells in IHD. Adipose-derived stem cells are a group of mesenchymal stem cells easy to harvest and with reduced immunogenicity. Molecular biology techniques aim at inducing differentiation into those cells, thus enhancing a potent paracrine signaling that aids in immunomodulation and angiogenesis or making them differentiate into cardiomyocytes, endothelial cells or pacemaker cells. Although promising results derived from some clinical trials, carefulness is needed in firmly supporting the use of such therapies in clinical practice, and further studies are warranted.

In conclusion, modern evidence is gradually assessing the pivotal role of inflammation in IHD, moving beyond the concept of atherosclerosis of a mere cholesterol excess-derived illness and embracing the theory of a broader inflammatory disease. The so-called “residual inflammatory risk” may in fact justify the excess of cardiovascular events even in those patients with strict (traditional) risk factors control. Further studies are therefore needed to deepen our knowledge about the correlation between inflammation and IHD in order to pave the way to new therapies aimed at improving the outcome and quality of life of patients who bear the burden of atherosclerotic heart disease.
